# A pilot study investigating early postoperative changes of plasma polyunsaturated fatty acids after laparoscopic sleeve gastrectomy

**DOI:** 10.1186/1476-511X-13-62

**Published:** 2014-04-03

**Authors:** Mutay Aslan, Ibrahim Aslan, Filiz Özcan, Ramazan Eryılmaz, Cemal Ozben Ensari, Tuna Bilecik

**Affiliations:** 1 Department of Medical Biochemistry, Akdeniz University Medical Faculty, Antalya, Turkey; 2 Endocrinology Clinic, Antalya Research and Education Hospital, Antalya, Turkey; 3 Surgery Clinic, Antalya Research and Education Hospital, Antalya, Turkey; 4 Department of Biochemistry, Akdeniz University Medical School, Antalya 07070, Turkey

**Keywords:** Laparoscopic sleeve gastrectomy, Polyunsaturated fatty acids, Insulin, Prostaglandin

## Abstract

**Background:**

This study aimed to determine early postoperative changes of plasma polyunsaturated fatty acids (PUFAs) following laparoscopic sleeve gastrectomy (LSG).

**Methods:**

Ten obese patients (mean BMI: 51.10 ± 11.59 kg/m^2^) underwent LSG and eleven normal weight control patients (mean BMI: 24.37 ± 2.33 kg/m^2^) underwent laparoscopic abdominal surgery. Fasting blood samples were collected prior to surgery, at day 1 after surgery and after postoperation oral feeding. Plasma levels of arachidonic acid (AA, C20:4n6), dihomo-gamma-linolenic acid (DGLA, C20:3n6), eicosapentaenoic acid (EPA, C20:5n3) and docosahexaenoic acid (DHA, C22:6n3) were determined by an optimized multiple reaction monitoring (MRM) method using ultra fast-liquid chromatography (UFLC) coupled with tandem mass spectrometry (MS/MS). Prostaglandin E_2_ (PGE2) was measured in serum samples by enzyme immunoassay.

**Results:**

A significant decrease was observed in insulin and HOMA IR levels in sleeve gastrectomy patients after postoperation oral feeding compared to preoperation. Plasma AA levels and AA/EPA ratio were significantly increased in sleeve gastrectomy patients after postoperation oral feeding compared to postoperation day 1. Serum PGE2 levels and AA/DHA ratio was significantly higher in sleeve gastrectomy patients at preoperation, postoperation day 1 and after postoperation oral feeding when compared to control group patients.

**Conclusion:**

Increased peripheral insulin sensitivity associated with LSG may play a role in the significant increase of plasma AA levels in sleeve gastrectomy patients following postoperation oral feeding. The significant increase in PGE2 levels and AA/DHA ratio in sleeve gastrectomy group patients also confirms the presence of a proinflammatory state in obesity.

## Introduction

The human body can produce many fatty acids except the two essential polyunsaturated fatty acids (PUFAs) which include linoleic acid (LA, C18:2n6) and alpha-linolenic acid (ALA, C18:3n3) [1]. Linoleic acid is the precursor of omega-6 (n-6) series of PUFAs while ALA is the precursor of omega-3 (n-3) series of PUFAs [2]. Eicosanoids derived from n-6 PUFAs such as arachidonic acid (AA, C20:4n6) have proinflammatory and immunoactive functions, whereas eicosanoids derived from n-3 PUFAs such as eicosapentaenoic acid (EPA, C20:5n3) and docosahexaenoic acid (DHA, C22:6n3) have anti-inflammatory properties, attributed to their ability to inhibit the formation of n-6 PUFA-derived eicosanoids [3]. Recent studies have documented the presence of an imbalance in PUFA levels and its correlation with visceral fat accumulation in male subjects [4]. Moreover, a correlation between acute phase proteins and serum PUFA composition was shown in morbidly obese patients [5].

Laparoscopic sleeve gastrectomy (LSG) is associated with a high rate of resolution of type 2 diabetes mellitus (T2DM) and other obesity-associated comorbidities such as hypertension and hyperlipidemia [6]. The improvement of insulin action occurs very early at 3–5 days following LSG with a significant reduction in insulin resistance [7]. Insulin stimulates the conversion of essential fatty acids (LA and ALA) to longer-chain PUFAs [8]. Indeed, levels of the principal n-6 PUFA, AA, are reported to be significantly lower in diabetic patients than in controls [9,10]. It was recently shown that insulin analog initiation therapy significantly increased plasma PUFA levels in patients with T2DM [11]. Restoration of the first phase of insulin secretion and improved insulin sensitivity in diabetic obese patients immediately after sleeve gastrectomy, before any weight loss, seem to be related to hormonal changes of possible gastric origin and is neither meal- nor weight-change-related [12]. To our knowledge no study has evaluated the effect of LSG on plasma levels of PUFAs. This study aimed to assess early postoperative effects of LSG on plasma n-6 and n-3 PUFA levels.

## Materials and methods

### Patients

#### Study groups

The control group included 11 patients who were admitted to Antalya Research and Education Hospital, Surgery Clinic. Patients in the control group underwent laparoscopic abdominal surgery for appendectomy (n = 5), cholecystectomy (n = 4), partial cystectomy (n = 1) and inguinal hernia repair (n = 1). Subjects with apparent history of stroke, coronary heart disease, arrhythmia, peripheral artery disease, severe kidney dysfunction, liver disease, thyroid dysfunction, infectious disease were excluded. The body mass index (BMI) of all patients in the control group was <30 kg/m^2^ and all were non-smokers. Fasting blood samples were obtained from all patients at preoperation, postoperation day 1 and after postoperation oral feeding.

The sleeve gastrectomy group included 10 obese patients who were admitted to Antalya Research and Education Hospital, Endocrinology Clinic. The BMI of all patients in the sleeve gastrectomy group was ≥40 kg/m^2^. All patients went through a clinical, biochemical and pre-anesthetic evaluation and subjects with apparent history of stroke, coronary heart disease, arrhythmia, peripheral artery disease, severe kidney dysfunction, liver disease, thyroid dysfunction, infectious disease were excluded. All patients met the surgical indication criteria in the inter-disciplinary European guidelines on surgery of severe obesity [13]. Fasting blood samples were obtained from all sleeve gastrectomy patients the day before operation (preoperation), the day after operation (postoperation day 1) and the day after postoperation oral feeding. All sleeve gastrectomy patients were on preoperative diet for 2 weeks, before surgery. This diet contained liquid protein supplements and sugar-free, non-carbonated, low calorie fluids and required a minimum of 2 liters of fluid intake daily. Female and male patients were aimed to receive 65 and 80 grams protein daily, respectively. Patients did not receive any food and no fluid by mouth starting from midnight the day of surgery until the day after surgery. Patients were tested several times after surgery for anastomatic leaks. When patients were determined to be leak free, postoperation oral feeding was initiated by aiming 120 ml per hour fluid intake. Clear bouillon, sugar-free gelatin, sugar-free flavored beverages, 1:1 water diluted apple, cranberry, or grape juice were allowed to be added to the diet at this stage. Postoperation oral feeding was continued until the patients were discharged from the hospital. All sleeve gastrectomy patients and control group subjects gave written informed consent prior to entry. This study was approved by the Institutional Review Board for Human Use at Akdeniz University Faculty of Medicine.

### Laboratory measurements

Serum glucose was measured on Roche Cobas 8000 Modular Analyser (Basel, Switzerland). Insulin levels were measured by Roche/Hitachi E170 modular analyser (Tokyo, Japan). Insulin sensitivity was evaluated using homeostatic model assessment for insulin resistance (HOMA IR) [14].

### Electrospray ionization mass spectrometry

Standards for AA (C20:4n6), DGLA (C20:3n6), EPA (C20:5n3) and DHA (C22:6n3) were purchased from Sigma-Aldrich (St. Louis MO, USA). Deuterium labeled AA-d8 internal standard (5,6,8,9,11,12,14,15-AA-d8) was obtained from Santa Cruz Biotechnology (Santa Cruz, CA, USA). Solutions of AA, DGLA, EPA, DHA and AA-d8 standards were prepared in analytical grade methanol (Merck, Darmstadt, Germany). An optimized multiple reaction monitoring (MRM) method was developed using ultra-fast liquid chromatography (UFLC) coupled with tandem mass spectrometry (MS/MS). A UFLC system (LC-20 AD UFLC XR, Shimadzu Corporation, Japan) was coupled to a LCMS-8040 triple quadrupole mass spectrometer (Shimadzu Corporation, Japan). Chromatographic separations were carried out using Inertsil HPLC column (ODS-4, 2.1 × 100 mm, 3 μm; GL Sciences Inc. Tokyo, Japan) maintained at 40°C. DHA, EPA, AA and DGLA were separated using a gradient elution with a flow rate of 0.45 ml/min. Mobile phase solvent A was 10 mM ammonium acetate (Sigma-Aldrich, St. Louis, MO, USA) in water and solvent B was acetonitrile (Sigma-Aldrich, St. Louis, MO, USA). Gradient program was solvent B, 70% (0 min), 90% (3 min), 100% (3.01-4 min) and 70% (4.01-8 min). MRM transitions and responses were automatically optimized for individual compounds in negative electrospray ionization (ESI). In the negative ESI-MS mode the precursor and product m/z values for AA, DHA, EPA, DGLA and AA-d8 are given in Table 1. Responses to AA, DHA, EPA and DGLA were optimized to a linear calibration range from 100 ng/ml to 30 ug/ml and a sample analysis time of 8 minutes.

**Table 1:** The precursor and product m/z values for analyzed polyunsaturated fatty acids

	**Precursor m/z**	**Product m/z**
**DGLA (C20:3n6)**	304.80	59.00, 260.70
**AA (C20:4n6)**	303.10	59.00, 258.90
**EPA (C20:5n3)**	301.10	59.10, 256.70
**DHA (C22:6n3)**	327.10	59.10, 283.20
**AA-d8**	311.10	59.10, 97.90, 267.10

DGLA, dihomo-gamma-linolenic acid; AA, arachidonic acid; EPA, eicosapentaenoic acid; DHA, docosahexaenoic acid.

### Sample preparation for LC-MS/MS

Samples were prepared for LC-MS/MS analysis via a modified protocol as previously described [15]. Briefly, in a glass test tube, 200 μl plasma was added to 200 μl AA-d8 internal standard solution. 1 ml of acetonitril/37% hydrochloric acid (Cayman, Ann Arbor, MI, USA) was added to the mixture in a 4:1 v/v. Tubes were capped with reusable teflon liner screw caps and samples were hydrolyzed by incubating at 90°C for 2 hours in a heating block (VLM, Bielefeld, Germany). After cooling down to room temperature, fatty acids were extracted with 2 ml of hexane. Samples were vortex-mixed for 20 seconds, left at room temperature for 5 minutes and centrifuged at 3000 rpm for 1 minute. The upper phase containing free fatty acids were transferred to glass tubes and evaporated at room temperature under a constant stream of nitrogen with height adjustable gas distribution unit (VLM, Bielefeld, Germany). Fatty acids were dissolved in 200 μl methanol–water (180:20, v/v) filtered via 0,2 μm polytetrafluoroethylene (PTFE) syringe filters (Whatman, GE Healthcare Bio-Sciences, Pittsburgh, USA) and transferred to autosampler vials (Vertical Chromatography, Nonthaburi, Thailand).

### Measurement of prostaglandin E_2_

Prostaglandin E_2_ (PGE2) was measured in serum samples by a commercial enzyme immunoassay test kit [KGE004B; R&D Systems, Inc., Minneapolis, MN 55413, USA] according to manufacturer’s instructions. A standard curve of absorbance values of known PGE2 standards was plotted as a function of the logarithm of PGE2 standard concentrations (pg/ml) using the GraphPad Prism Software program for windows version 5,03. (GraphPad Software Inc). PGE2 concentrations in the samples were calculated from their corresponding absorbance values via the standard curve.

### Statistical analysis

Data were analyzed using Sigma Stat (version 2.03) statistical software for Windows, and a P value < 0.05 was considered statistically significant.

## Results

### Control and sleeve gastrectomy group characteristics

The control group was composed mainly of women (7 female, 4 male). The mean ± SD of age, body weight and body mass index in the control group was 41 ± 18 years, 65 ± 6 kg and 24.37 ± 2.33 kg/m^2^, respectively. The sleeve gastrectomy group was also composed mainly of women (7 female, 3 male). The mean ± SD of age, body weight, body mass index, fat mass and lean mass in the sleeve gastrectomy group was 38 ± 11 years, 130 ± 22 kg, 51.10 ± 11.59 kg/m^2^, 67.2 ± 15 kg and 59.7 ± 10.3 kg respectively.

### Biochemical measurements

Glucose, insulin and HOMA IR levels from control and sleeve gastrectomy patients are shown in Table 2. A significant decrease was observed in insulin and HOMA IR levels in sleeve gastrectomy patients after postoperation oral feeding compared to preoperation. Statistical analysis was done by Paired t-test.

**Table 2:** Serum glucose, insulin concentration and HOMA-IR values in control and sleeve gastrectomy group

**Control group**			**Sleeve gastrectomy group**	
**Variable**	**Preop (n = 11)**	**Po OF (n = 11)**	**Preop (n = 10)**	**Po OF (n = 10)**
**Glucose (mg/dL)**	103.0 ± 21.6	96.9 ± 22.7	102.2 ± 26.8	102.5 ± 31.3
**Insulin (mU/L)**	9.7 ± 9.4	7.7 ± 6.9	18.8 ± 10.2	11.1 ± 6.9*
**HOMA-IR**	2.7 ± 3.3	2.1 ± 2.9	4.9 ± 3.5	2.9 ± 2.1*

Values are mean ± SD. Preop, preoperation; Postop, postoperation; Po OF, postoperation oral feeding. *p < 0.01 postoperation oral feeding vs. preoperation in the sleeve gastrectomy group.

### ESI-MS spectra

**Figure 1:**
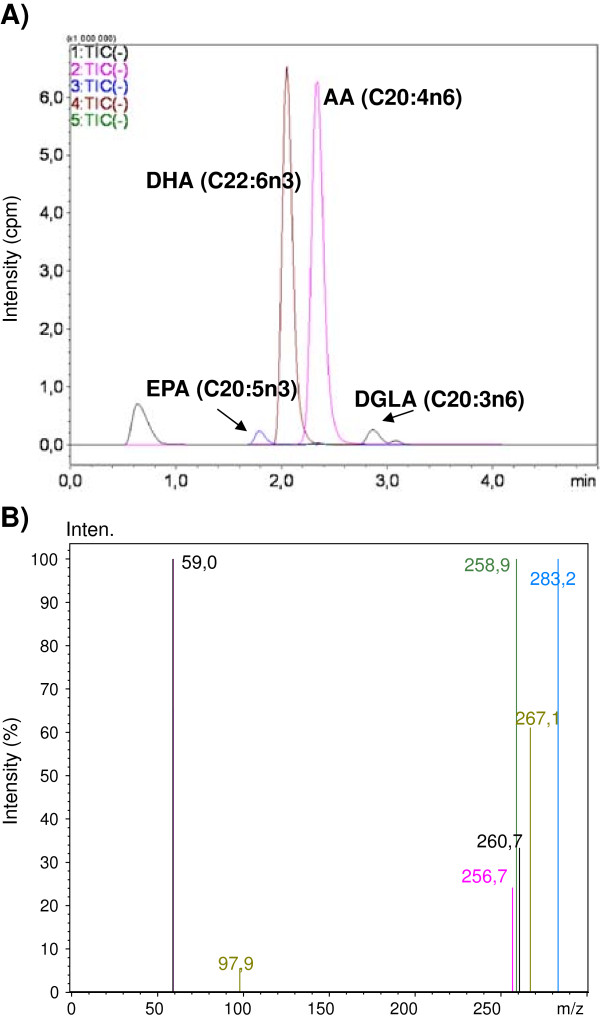
**ESI-MS Spectra. A)** Representative negative ion mode spectra of a patient sample. DGLA, Dihomo-gamma-linolenic acid; AA, Arachidonic acid; EPA, Eicosapentaenoic acid; DHA, Docosahexaenoic acid. **B)** Tandem mass spectra.

Figure 1A shows representative negative ion mode spectra of a patient sample. As shown in the figure, retention time of EPA (C20:5n3), DHA (C22:6n3), AA (C20:4n6) and DGLA (C20:3n6) was 1.869, 2.131, 2.391 and 2.911 minutes, respectively. Figure 1B shows tandem mass spectra obtained by collision-induced dissociation of precursor ions. The m/z values of 256.7, 258.9, 260.7, 283.2 product ions correspond to endogenous C20:5n3, C20:4n6, C20:3n6 and C22:6n3, respectively. The deuterium-labeled internal standard fatty acid peaks are indicated at m/z values 97.9 and 267.1.

### Levels of polyunsaturated fatty acids

**Figure 2:**
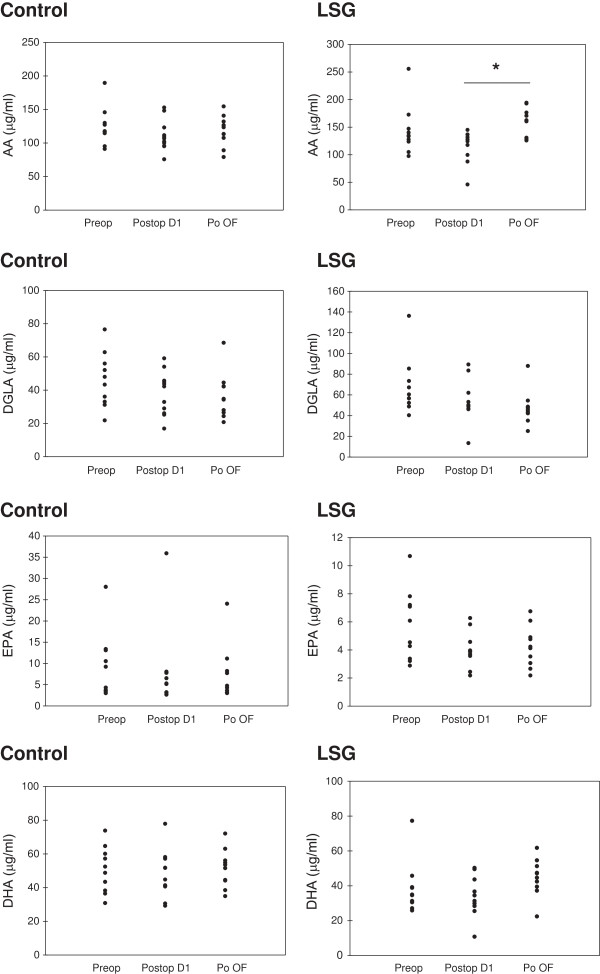
**Distribution of measured polyunsaturated fatty acids in control and sleeve gastrectomy patients from preoperation up to postoperation oral feeding.** AA, Arachidonic acid; DGLA, Dihomo-gamma-linolenic acid; EPA, Eicosapentaenoic acid; DHA, Docosahexaenoic acid.

Blood samples obtained from control group patients showed no significant difference in levels of AA (C20:4n6), DGLA (C20:3n6), EPA (C20:5n3) and DHA (C22:6n3) (Table 3). The ratio of DGLA/AA, AA/EPA and AA/DHA were also similar in control group samples obtained at preoperation, postoperation day 1 and after postoperation oral feeding. Likewise, DGLA (C20:3n6), EPA (C20:5n3) and DHA (C22:6n3) serum levels in sleeve gastrectomy patients were similar at preoperation, postoperation day 1 and after postoperation oral feeding. There was also no significant difference observed in DGLA (C20:3n6), EPA (C20:5n3) and DHA (C22:6n3) serum levels between control and sleeve gastrectomy patients (Table 3). Serum AA (C20:4n6) levels and AA/EPA ratio were significantly increased in sleeve gastrectomy patients after postoperation oral feeding compared to postoperation day 1. Levels of AA (C20:4n6) and AA/EPA ratio were also significantly higher in sleeve gastrectomy patients after postoperation oral feeding compared to control group patients on the same day. AA/DHA ratio was significantly higher in sleeve gastrectomy patients at preoperation, postoperation day 1 and after postoperation oral feeding when compared to control group patients (Table 3). Statistical analysis was done by Paired t-test or Wilcoxon Signed Rank Test. Distribution of AA (C20:4n6), DGLA (C20:3n6), EPA (C20:5n3) and DHA (C22:6n3) in serum samples of control and sleeve gastrectomy patients are shown in Figure 2.

**Table 3:** Analysis of polyunsaturated fatty acids in control and sleeve gastrectomy group from preoperation up to postoperation oral feeding

**Control group**				**Sleeve gastrectomy group**		
**Variable**	**Preop (n = 11)**	**Postop day 1 (n = 11)**	**Po OF (n = 11)**	**Preop (n = 10)**	**Postop day 1 (n = 10)**	**Po OF (n = 10)**
**AA (C20:4n6) (μg/ml)**	124.0 ± 27.8	110.5 ± 22.8	117.5 ± 22.7	143.1 ± 44.5	114.7 ± 30.0	159.7 ± 25.0*,^a^
**DGLA (C20:3n6) (μg/ml)**	45.9 ± 16.4	37.9 ± 13.1	36.4 ± 13.8	67.4 ± 27.2	53.9 ± 21.0	47.1 ± 16.3
**EPA (C20:5n3) (μg/ml)**	9.1 ± 7.7	7.9 ± 9.4	7.2 ± 6.4	5.6 ± 2.5	4.0 ± 1.2	4.2 ± 1.4
**DHA (C22:6n3) (μg/ml)**	50.4 ± 13.6	47.4 ± 13.8	51.1 ± 11.2	38.3 ± 14.8	33.8 ± 11.8	44. 6 ± 10.6
**DGLA/AA**	0.38 ± 0.14	0.34 ± 0.11	0.31 ± 0.11	0.47 ± 0.13	0.46 ± 0.12	0.31 ± 0.15**
**AA/EPA**	23.7 ± 18.1	23.3 ± 12.9	24.7 ± 12.6	28.2 ± 11.9	29.2 ± 5.6	42.0 ± 16.1**,^a^
**AA/DHA**	2.64 ± 0.94	2.45 ± 0.65	2.39 ± 0.63	3.87 ± 0.85^a^	3.54 ± 0.66^a^	3.84 ± 1.50^a^

Values are mean ± SD. Preop, preoperation; Postop, postoperation; Po OF, postoperation oral feeding; DGLA, Dihomo-gamma-linolenic acid; AA, Arachidonic acid; EPA, Eicosapentaenoic acid; DHA, Docosahexaenoic acid.

*p = 0.007 postoperation oral feeding vs. postoperation day 1 in the sleeve gastrectomy group.

**p < 0.02 postoperation oral feeding vs. preoperation and postoperation day 1 in the sleeve gastrectomy group.

^a^p < 0.02 sleeve gastrectomy group vs. control (the same day).

### Prostaglandin E_2_ levels

**Figure 3:**
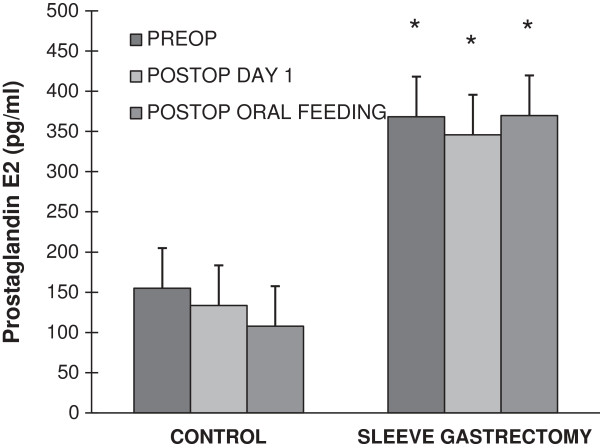
**Prostaglandin E**_**2 **_**in control and sleeve gastrectomy group from preoperation up to postoperation oral feeding.** *p < 0.05 sleeve gastrectomy group vs. control (the same day).

Bar graph data of serum PGE2 content are shown in Figure 3. Serum PGE2 levels were similar at preoperation, postoperation day 1 and after postoperation oral feeding in the control group. Likewise, no significant difference was observed in serum PGE2 levels within sleeve gastrectomy patients. Serum PGE2 levels were significantly higher in sleeve gastrectomy patients at preoperation, postoperation day 1 and after postoperation oral feeding when compared to control group patients (Figure 3). Statistical analysis was done by paired t-test.

## Discussion

Polyunsaturated fatty acids regulate inflammatory responses through the production of eicosanoids including prostaglandins (PGs), thromboxanes (TXs) and leukotrienes (LTs) [2]. To our knowledge, this is the first study evaluating early postoperative effects of LSG on plasma PUFA levels. Plasma AA (C20:4n6) levels and AA/EPA ratio were significantly increased in sleeve gastrectomy patients after postoperation oral feeding compared to postoperation day 1. The observed significant increase of plasma AA levels following LSG may be due to increased peripheral insulin sensitivity. In agreement with previous studies [7,16] we have observed a significant reduction in insulin levels occurring very early at 4–5 days following sleeve gastrectomy with a significant reduction in insulin resistance. It was recently shown that insulin initiation therapy significantly increased plasma levels of AA (C20:4n6) compared to before treatment levels in T2DM patients [11]. Disturbed fatty acid metabolism is an important feature of the insulin-resistant state [17]. Essential fatty acids are metabolized into more physiologically active compounds by introduction of further double bonds by delta-5- and delta-6-desaturase enzymes [9]. Emerging evidence shows that delta-5 desaturase is the key regulator in the synthesis of PUFA and is modulated by factors including adiposity, diet and insulin resistance [18]. The hepatic microsomal delta-6-desaturation of LA and ALA was found to be depressed in alloxan induced diabetic rats [19]. The observed enzymatic defect was corrected by insulin injection in 2 days [20].

High levels of saturated fatty acids and low amounts of PUFAs are associated with obesity [21]. Omega-3 PUFA enriched diet increases expression of genes involved in glucose transport [glucose transporter type 4 (GLUT-4)] and insulin signaling [insulin receptor substrate 1 (IRS-1)], as well as genes involved in insulin sensitivity [peroxisome proliferator-activated receptor gamma (PPARγ)] [22]. In an insulin-resistant state, omega-3 PUFAs bind to the G-protein coupled receptor 120 (GPR120), resulting in reduced cytokine production from inflammatory macrophages and improved signaling in adipocytes, leading to a reduction in insulin resistance [23]. In this study, we have observed a significant increase in AA/EPA ratio in sleeve gastrectomy patients after postoperation oral feeding compared to preoperation levels. This finding suggests that improved insulin sensitivity in obese patients immediately after sleeve gastrectomy in not related to changes in omega-3 PUFA levels.

Low serum EPA/AA ratio was recently reported in male subjects with visceral obesity [4]. Likewise, an imbalance of dietary long-chain PUFAs, especially high omega-6/omega-3 PUFA ratio, was associated with increased risk of cardiovascular disease [24]. We have observed that AA (C20:4n6)/DHA(C22:6n3) ratio was significantly higher in sleeve gastrectomy patients at preoperation, postoperation day 1 and after postoperation oral feeding when compared to control group subjects. The significant increase of PGE2 levels and AA/DHA ratio in all performed measurements in the sleeve gastrectomy group patients supports the presence of a proinflammatory state in obesity. Competition between omega-6 and omega-3 fatty acids occurs in the production of eicosanoids by stereospecific lipid-oxidizing enzymes cylooxygenase (COX) and lipoxygenase (LOX) [25]. Docosahexaenoic acid (DHA, C22:6n3) is a precursor of eicosanoids with less marked inflammatory effect. On the other hand, AA is a precursor of eicosanoids with definite inflammatory effect [26]. Hence, increased AA to DHA ratio indicates more precursor for the synthesis of highly inflammatory eicosanoids.

As stated in the introduction of the manuscript, restoration of the first phase of insulin secretion and improved insulin sensitivity in diabetic obese patients immediately after sleeve gastrectomy, before any weight loss, seem to be related to hormonal changes of possible gastric origin and is neither meal- nor weight-change-related [12]. Significantly increased AA levels in sleeve gastrectomy patients after postoperation oral feeding may play a constructive role in the observed improved insulin sensitivity in sleeve gastrectomy patients. It was previously observed that patients with type 2 diabetes mellitus have low AA content in their plasma phospholipid fraction and it was suggested that AA deficiency may predispose humans to develop T2DM [27]. In support of these findings it was recently demonstrated that insulin analog initiation therapy in T2DM patients increased AA in human plasma (11). The role of AA as a possible endogenous anti-diabetic molecule is discussed comprehensively in a recent review [28].

Limitation of our study include: 1) the study covers a small cohort; 2) the study has a nonrandomized design because patients were assigned to either group according to clinical criteria; 3) The study population included an unequal percentage of male patients. However, the limitation of gender distribution has been minimized by matching the control group similarly.

In summary, we have observed a significant decrease in insulin and HOMA IR levels in sleeve gastrectomy patients after postoperation oral feeding compared to preoperation. This was accompanied by significantly increased plasma AA levels. The significant increase in PGE2 levels and AA/DHA ratio in sleeve gastrectomy group patients also confirms the presence of a proinflammatory state in obesity.

## Abbreviations

AA: Arachidonic acid; ALA: Alpha-linolenic acid; COX: Cylooxygenase; DHA: Docosahexaenoic acid; EPA: Eicosapentaenoic acid; ESI: Electrospray ionization; GLUT-4: Glucose transporter type 4; GPR120: G-protein coupled receptor 120; IRS-1: Insulin receptor substrate 1; LA: Linoleic acid; LOX: Lipoxygenase; LSG: Laparoscopic sleeve gastrectomy; LTs: Leukotrienes; omega-3: n-3; omega-6: n-6; PGE2: Prostaglandin E_2_; PGs: Prostaglandins; PPARγ: Peroxisome proliferator-activated receptor gamma; PTFE: Polytetrafluoroethylene; PUFAs: Polyunsaturated fatty acids; T2DM: Type 2 diabetes mellitus; TXs: Thromboxanes.

## Competing interests

All authors declare that they have no financial, consulting, and personal relationships with other people or organizations that could influence the presented work.

## Authors’ contributions

MA, carried out LC-MS/MS analysis, measurement of PGE2 and drafted the manuscript. FO, carried out LC-MS/MS analysis and measurement of PGE2. IA, carried out the clinical studies including enrollment of patients, collection of blood samples and contributed in the drafting of the manuscript. RE, carried out the clinical studies including enrollment of patients, collection of blood samples. COE, carried out the clinical studies including enrollment of patients, collection of blood samples. TB, carried out the clinical studies including enrollment of patients, collection of blood samples. All authors read and approved the final manuscript.
